# The role of the genomic mutation signature and tumor mutation burden on relapse risk prediction in head and neck squamous cell carcinoma after concurrent chemoradiotherapy

**DOI:** 10.1038/s12276-023-00984-4

**Published:** 2023-05-01

**Authors:** Hui-Ching Wang, Sin-Hua Moi, Leong-Perng Chan, Chun-Chieh Wu, Jeng-Shiun Du, Pei-Lin Liu, Meng-Chun Chou, Che-Wei Wu, Chih-Jen Huang, Hui-Hua Hsiao, Mei-Ren Pan, Li-Tzong Chen

**Affiliations:** 1 Graduate Institute of Clinical Medicine, College of Medicine, Kaohsiung Medical University, Kaohsiung, 807 Taiwan; 2 Department of Internal Medicine, Division of Hematology and Oncology, Kaohsiung Medical University Hospital, Kaohsiung Medical University, Kaohsiung, 807 Taiwan; 3 Faculty of Medicine, College of Medicine, Kaohsiung Medical University, Kaohsiung, 807 Taiwan; 4 Research Center for Precision Environmental Medicine, Kaohsiung Medical University, Kaohsiung, 807 Taiwan; 5 Department of Otolaryngology-Head and Neck Surgery, Kaohsiung Medical University Hospital, Kaohsiung Medical University, Kaohsiung, 807 Taiwan; 6 Department of Pathology, Kaohsiung Medical University Hospital, Kaohsiung Medical University, Kaohsiung, 807 Taiwan; 7 Department of Nursing, Kaohsiung Medical University Hospital, Kaohsiung Medical University, Kaohsiung, 807 Taiwan; 8 Department of Radiation Oncology, Kaohsiung Medical University Hospital, Kaohsiung Medical University, Kaohsiung, 807 Taiwan; 9 Drug Development and Value Creation Research Center, Kaohsiung Medical University, Kaohsiung, 807 Taiwan; 10 Department of Medical Research, Kaohsiung Medical University Hospital, Kaohsiung Medical University, Kaohsiung, 807 Taiwan; 11 National Institute of Cancer Research, National Health Research Institutes, Tainan, Taiwan; 12 Center for Cancer Research, Kaohsiung Medical University, Kaohsiung, 807 Taiwan

**Keywords:** Genome-wide association studies, Oral cancer

## Abstract

Personalized genetic profiling has focused on improving treatment efficacy and predicting risk stratification by identifying mutated genes and selecting targeted agents according to genetic testing. Therefore, we evaluated the role of genetic profiling and tumor mutation burden (TMB) using next-generation sequencing in patients with head and neck squamous cell carcinoma (HNSC). The relapse mutation signature (RMS) and chromatin remodeling mutation signature (CRMS) were explored to predict the risk of relapse in patients with HNSC treated with concurrent chemoradiotherapy (CCRT) with platinum-based chemotherapy. Patients in the high RMS and CRMS groups showed significantly shorter relapse-free survival than those in the low RMS and CRMS groups, respectively (*p* < 0.001 and *p* = 0.006). Multivariate Cox regression analysis showed that extranodal extension, CCRT response, and three somatic mutation profiles (TMB, RMS, and CRMS) were independent risk predictors for HNSC relapse. The predictive nomogram showed satisfactory performance in predicting relapse-free survival in patients with HNSC treated with CCRT.

## Head and neck cancer: genetic profiling improves predictions of relapse

Including personalized genetic profiling data greatly improves calculations of the risk of relapse in head and neck squamous cell cancer (HNSCC) after treatment. HNSCC, which develops in the nose, mouth and throat, has a 5-year overall survival rate of ~50% due to recurrence. Mei-Ren Pan and Li-Tzong Chen at Kaohsiung Medical University in Taiwan and co-workers analyzed genetic, clinical and survival data from 120 HNSCC patients who had received a course of concurrent radiotherapy and chemotherapy. The researchers developed a computational model that revealed key genetic risk predictors, such as the tumor mutation burden, the total number of mutations in the cancer cell DNA. The model showed much better performance in predicting relapse-free survival than one based solely on clinical and laboratory data, indicating the huge potential benefits of including genetic profiles in prognostic models.

## Introduction

Head and neck squamous cell carcinoma (HNSC) is the sixth most common cancer worldwide. Despite significant progress in all therapeutic modalities, such as surgery, radiotherapy, and chemotherapy, the 5-year overall survival (OS) rate for patients with HNSC is ~50%, and the main reason for treatment failure is the frequent development of locoregional recurrence^1^. Fifty to 60% of patients receiving front-line treatment develop locoregional recurrence within 2 years. Approximately 20–30% of these patients develop distant metastases^2^. Recently, a phase 2 study with Debio 1143, an orally available inhibitor of apoptotic proteins, in patients with locally advanced HNSC achieved superior efficacy against a high-dose cisplatin chemoradiotherapy comparator in a randomized trial^3^. Improvement of treatment outcomes and disease control are mandatory in HNSC.

Several clinical factors have been linked to recurrence after front-line treatment, such as human papillomavirus (HPV) infection^4^, positive surgical margins^5^, extranodal extension (ENE)^6,7^, multiple cervical lymph nodes^8^, inadequate radiation dose to the primary treatment volume^9^, and poor histologic differentiation of the primary tumor^10^. Some of these patients are defined as high-risk patients with HNSC. To decrease the further risk of recurrence and improve survival, concurrent chemoradiotherapy (CCRT) combining radiotherapy and high-dose platinum demonstrated better outcomes than conventional radiotherapy alone^5,7^. In addition to clinical factors, some genomic alterations also demonstrated predictive value for clinical outcomes in HNSC. In HPV-negative HNSC, a 15-gene hypoxia classifier could identify patients with improved outcomes after combination radiotherapy^11^. Ten genes, forming an intrinsic radiosensitivity index, were evaluated as prognostic prediction models for locoregional control of chemoradiation in HNSC^12^. In Taiwan, a nine-gene OCSCC panel (*RYR1, HLA-B, TSHZ2, PCDH17, DNAH17, GRID1, SBNO2, KSR2*, and *GCN1L1*) was identified via whole-exome sequencing (WES) for the prediction of survival in 168 surgically treated patients with oral cancer^13^. Patients with alterations in the NOTCH, RTK/RAS/MAPK, and TGF-beta signaling pathways demonstrated poor disease-free survival^14^. In 151 French patients with oral cancer, transforming growth factor-β pathway alterations were associated with poor OS clinical outcomes using an in-house targeted next-generation sequencing (NGS) panel. High TMB was associated with prolonged OS in both the highest 10% and 20% TMB values^15^. These results revealed that analyses of genomic alterations and signaling pathways contribute to predicting patients’ clinical relevance.

CCRT, including radiotherapy and platinum-based chemotherapy, triggers a DNA damage response (DDR) through DNA strand breaks and the formation of DNA crosslinking products^16^. The DNA repair pathway plays an important role in maintaining genomic integrity and preventing DNA damage. High expression of the DNA repair pathway is associated with metastasis and drug resistance in various malignancies^17^. DNA repair pathways, including single-strand damage repair and double-strand break (DSB) repair, are closely related to regulating responses to chemoradiotherapy in patients with locally advanced HNSC. Interestingly, chromatin plays an important role in regulating DNA-associated processes; however, chromatin is also vulnerable to DNA damage. Chromatin interferes with accessibility to DNA and the structure of DNA and provides binding sites for associated proteins via the regulation of posttranslational histone modifications and nucleosome remodeling^18^. This relationship strongly suggests that alteration of chromatin remodeling may be linked to the response to DNA damage-associated therapeutic strategies.

Next-generation sequencing discloses cancer genomic information at different depths and levels. More cancer genomic knowledge, including somatic mutations, copy number variation, and tumor mutation burdens, is gradually being uncovered and linked with prognosis and treatment response. However, there are still many unknown areas to explore regarding the associations between the cancer genomic landscape and clinical outcomes. This study aimed to decipher the predictive value of a computational model by organizing clinicopathological factors, tumor mutation burdens, and genomic mutation signatures in patients with locally advanced HNSC treated with CCRT.

## Methods

### Data source

**Fig. 1:**
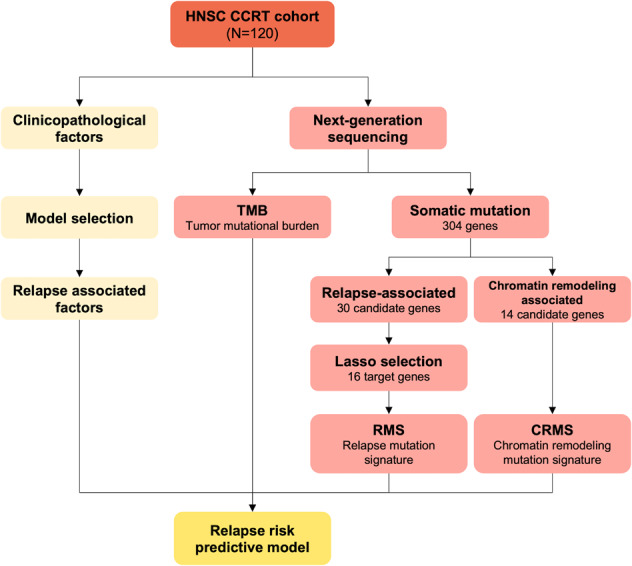
Analysis flowchart. HNSC: head and neck cancer, CCRT, concurrent chemoradiation therapy. RFS relapse-free survival. TMB tumor mutational burden.

In this retrospective cohort study, all data were retrospectively collected via the health information system of Kaohsiung Medical University Hospital under an approved protocol (KMUHIRB-E(I)-20210401). This study included patients with HNSC who had received CCRT. In total, 120 patients who met the inclusion criteria were included in the HNSC concurrent chemoradiotherapy (CCRT) group as the derivation cohort. The inclusion criteria were as follows: age at diagnosis ≥20 years; tumor histology of squamous cell carcinoma (grade 1 to grade 3); ICD-9 site code specific for the oral cavity (OC), hypopharynx (HPC), oropharynx (OPC), or larynx; and treatment with concurrent chemoradiotherapy (CCRT) with platinum-based chemotherapy. Eighty-four patients underwent surgical resection, and 36 patients underwent only tissue biopsy. Relapse-free survival (RFS) was considered the primary prognostic outcome. Patients with HNSC with relapse events within the study period were regarded as relapse cases, and those who were free of relapse events were regarded as controls. The clinicopathological factors and gene mutation characteristics of the derivation cohort were used to construct a relapse risk predictive model for the study cohort. A comprehensive analysis workflow is shown in Fig. 1. Clinicopathological factors included age, sex, tumor location, grade, margin, ENE, lymph node invasion (LVI), perineural invasion (PNI), pathological stage, surgery, induction, response to CCRT, relapse status, and overall survival status. Demographic data, clinical characteristics, laboratory findings, genomic information, and survival data were acquired from electronic medical records.

### Clinical review

Patient medical records were assessed for demographics, pathological features, induction and adjuvant therapy received, chemotherapy dosage, radiation dose and fractions, time of recurrence, and outcomes. RFS time was defined as the time from surgery for primary head and neck cancer until the diagnosis of tumor relapse. Clinical parameters were collected as follows: age (<45/45–64/>65 years), sex (male/female), primary site of tumor (hypopharynx/larynx/oral cavity/oropharynx), American Joint Committee on Cancer (AJCC) stage (I/II/III/IV), margin invasion (negative/positive), extranodal extension (absent/present), lymphovascular invasion (absent/present), perineural invasion (absent/present), and histological differentiation (grade 1/2/3). Pathological tumor stage (I/II/III/IV) was classified according to the eighth TNM (tumor-node-metastasis) staging edition.

The treatment response of patients was evaluated using RECIST 1.1-measurable lesions and classified into four categories: complete response (CR), partial response (PR), progressive disease (PD), and stable disease (SD), as reported in a previous study^19^. After disease progression, further treatments and survival status were documented every 3 months.

### Somatic mutation profiles

**Fig. 2:**
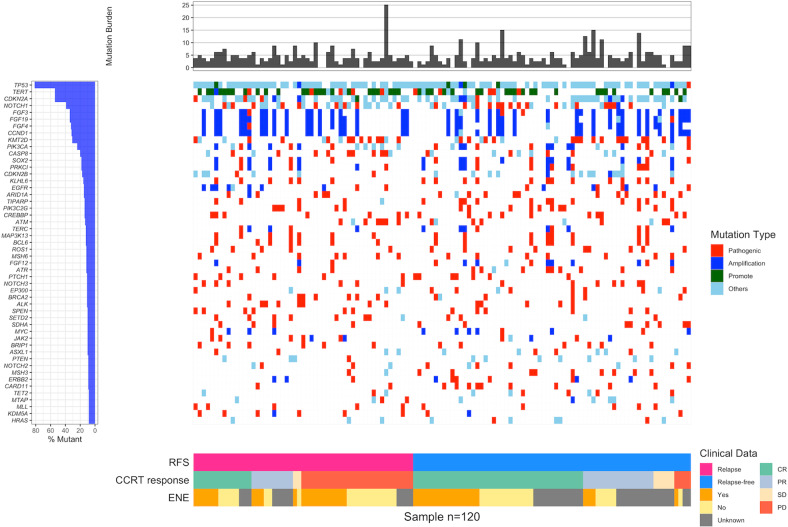
Somatic mutation profile for the top 50 mutated genes of the HNSC CCRT cohort according to next-generation sequencing results. Mutation burden indicates the tumor mutational burden (TMB, mut/Mb) of each patient.

**Fig. 3:**
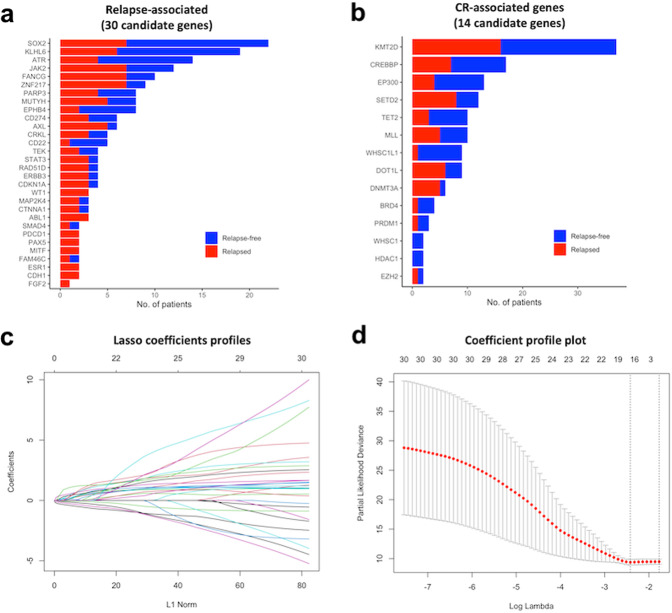
Relapse mutation signature (RMS) derived using LASSO regression and chromatin remodeling signature in the study population. **a** Bar plot shows the mutation rate of 30 relapse-associated candidate genes and (**b**) 14 chromatin remodeling-associated candidate genes. **c** LASSO coefficient profiles of mutated genes and (**d**) coefficient profile plot used to determine the optimal lambda for LASSO regression according to 30 relapse-associated candidate genes.

The somatic mutation profiles, including TMB and somatic mutations, were extracted from the medical records of the study cohort. Somatic mutations were detected by NGS, FoundationOne CDx (F1CDx), according to the Illumina® HiSeq 4000 platform, using 120 FFPE HNSC tissue specimens. The method of the F1CDx-targeted NGS platform has been validated previously^20^. TMB indicates the number of genetic mutations in cancer cells using mut/kB. There were 324 gene alterations detected via this assay, including all coding exons from 309 cancer-related genes; one promoter region; one noncoding RNA (ncRNA); and selected intronic regions from 34 commonly rearranged genes, 21 of which also included coding exons. The somatic mutation profile for the top 50 mutated genes and the corresponding TMB of the derivation cohort, according to NGS, are illustrated in Fig. 2. Next, mutated genes associated with RFS and chromatin remodeling were selected to construct a corresponding mutational signature, namely, RMS and CRMS. The mutation rates of 30 relapse-associated candidate genes and 14 chromatin remodeling-associated candidate genes according to relapse status are illustrated using a bar plot (Fig. 3a, b).

### Relapse mutation signature (RMS)

**Fig. 4:**
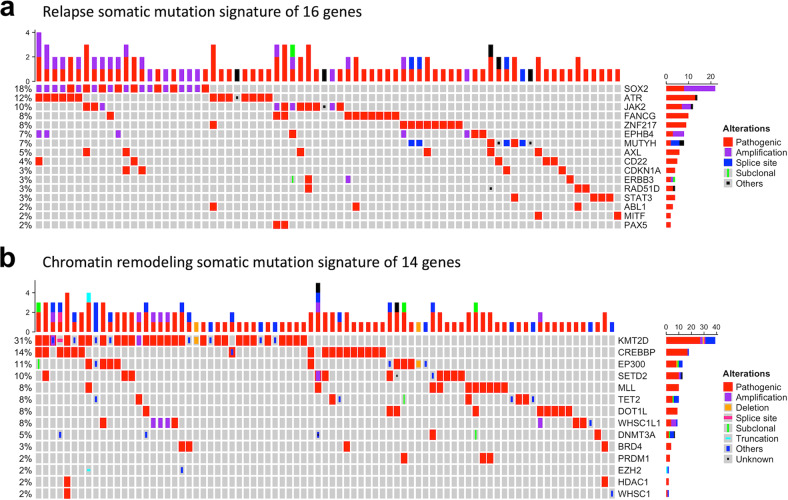
OncoPrint of the somatic mutational signatures for the HNSC CCRT cohort. (**a**) the relapse somatic mutation signature of 16 target genes and (**b**) the chromatin remodeling somatic mutational signature of 14 genes.

All somatic mutation genes were individually tested using the log-rank test according to RFS. The somatic mutation rate and RFS analysis results using the log-rank test for all the detected genes in the study cohort are summarized in Supplementary Table 1. Overall, 30 candidate genes with significant RFS differences between the mutation and wild type were selected to enter the LASSO regression model. The LASSO coefficient profiles (Fig. 3c) show the coefficient profile variation for each candidate gene according to the L1 normalization process in the LASSO regression model. Furthermore, the coefficient profile plot (Fig. 3d) was used to estimate the optimal gene combination for RFS prediction, derived using 30 relapse-associated candidate genes. Finally, 16 relapse-associated target genes were determined using the optimal lambda for LASSO regression, according to the 30 candidate genes. The somatic mutation signature of the 16 relapse-associated target genes was visualized using OncoPrint (Fig. 4a). Multivariate Cox regression analysis for the 16 target genes selected by LASSO regression was performed, and the regression coefficients (*β*) of each gene were computed (Table 2). RMS was generated using Cox regression *β* and mutation status (mut) of 16 relapse-associated target genes (*rg*). The equation for RMS is shown in Eq. (1).

1 $$RMS = \mathop {\sum }\limits_{rg = 1}^n \beta _{rg} \times mut_{rg},mut_{rg} = \left\{ {\begin{array}{*{20}{c}} {0 = wild} \\ {1 = mutated} \end{array}} \right.$$

### Chromatin remodeling mutation signature (CRMS)

Fourteen chromatin remodeling genes were identified in the study cohort. The somatic mutation signature of 14 chromatin remodeling-associated genes was visualized using OncoPrint (Fig. 4b). Multivariate Cox regression analysis for the 14 chromatin remodeling-associated genes was estimated, and the regression *β* of each gene was computed (Table 2). CRMS was generated using Cox regression *β* and mutation status (mut) of the 14 chromatin remodeling candidate genes (*crg*). The equation for CRMS is shown in Eq. (2).

2 $$CRMS = \mathop {\sum }\limits_{crg = 1}^n \beta _{crg} \times mut_{crg},mut_{crg} = \left\{ {\begin{array}{*{20}{c}} {0 = wild} \\ {1 = mutated} \end{array}} \right.$$

### Statistical analysis

**Fig. 5:**
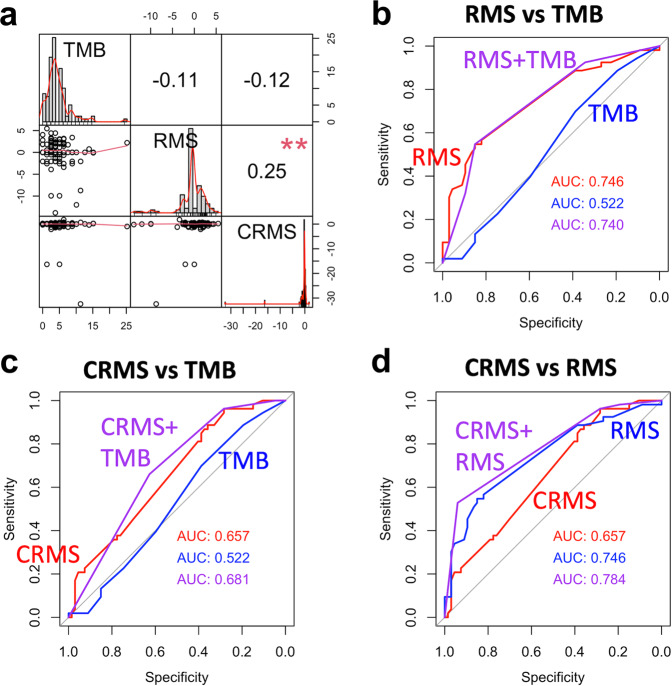
The correlation and satisfactory predictive performance of tumor mutational burden (TMB, mut/Mb), relapse mutation signature (RMS), and chromatin remodeling mutational signature (CRMS). **a** Correlation analysis results for TMB, RMS and CRMS. Satisfactory predictive performance comparison between (**b**) RMS and TMB and (**c**) CRMS and TMB and (**d**) relapse-free survival using ROC analysis. AUC area under the curve.

**Fig. 6:**
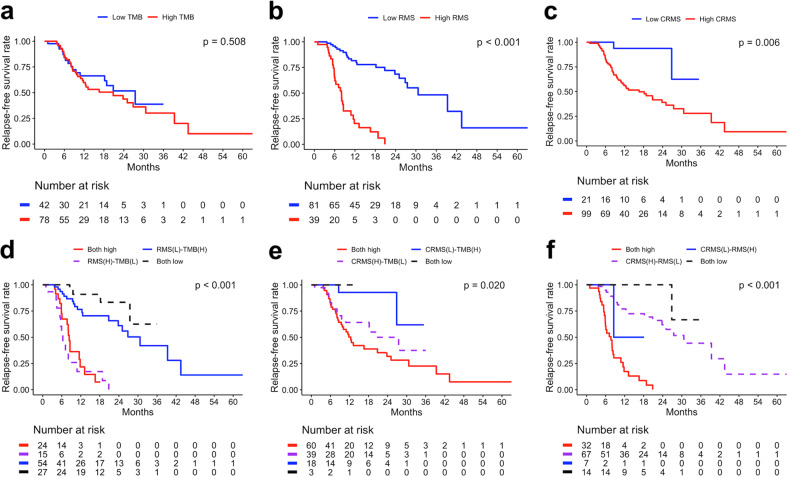
The prognostic risk predictive ability of TMB, RMS and CRMS subgroups for the HNSC CCRT cohort. Kaplan‒Meier plot for the (**a**) TMB subgroup, (**b**) RMS subgroup, (**c**) CRMS subgroup, (**d**) RMS-TMB subgroup, (**e**) CRMS-TMB subgroup, and (**f**) RMS-CRMS subgroup.

The baseline characteristics and TMB of the HNSC CCRT cohort are summarized as frequencies and percentages, and the distribution was estimated using the chi-square test or Fisher’s exact test. In addition, the continuous TMB is summarized as the median and interquartile range and was tested using the Wilcoxon rank sum test (Table 1). The distributions of TMB, RMS, and CRMS are presented using a histogram, and the correlation between TMB, RMS, and CRMS is shown using a scatter plot and was tested by the Pearson correlation test (Fig. 5a). The correlation coefficients between TMB, RMS, and CRMS were calculated. The satisfactory predictive performance of individual and pairwise interactions between TMB, RMS, and CRMS was estimated using receiver operating characteristic (ROC) analysis, and the area under the ROC curve (AUC) was reported (Fig. 5b–d). A higher AUC indicated better predictive performance. Furthermore, TMB, RMS, and CRMS were dichotomized into high and low subgroups using the optimal cutoff value derived using ROC analysis. The individual and pairwise interacting prognostic risk predictive ability among TMB, RMS, and CRMS for the study cohort was determined using the Kaplan‒Meier estimator and tested by log-rank test (Fig. 6). The prognostic risk predictive performance of TMB, RMS, CRMS, and the corresponding pairwise interaction combinations were further validated using the TCGA-HNSC validation cohort.

**Table 1:** Baseline characteristics of the HNSC CCRT derivation cohort (*n* = 120).

Characteristics	Overall, *n* = 120	Controls, *n* = 67	Relapse, *n* = 53	*P*
Age group (years)				0.527
<45	8 (6.7%)	3 (4.5%)	5 (9.4%)	
>65	35 (29.2%)	21 (31.3%)	14 (26.4%)	
45–64	77 (64.2%)	43 (64.2%)	34 (64.2%)	
Sex				0.731
Female	8 (6.7%)	4 (6.0%)	4 (7.5%)	
Male	112 (93.3%)	63 (94.0%)	49 (92.5%)	
Location				0.497
HPC	13 (10.8%)	6 (9.0%)	7 (13.2%)	
LC	4 (3.3%)	1 (1.5%)	3 (5.7%)	
OC	79 (65.8%)	45 (67.2%)	34 (64.2%)	
OPC	24 (20.0%)	15 (22.4%)	9 (17.0%)	
Grade				0.858
Grade 1	33 (28.2%)	19 (29.7%)	14 (26.4%)	
Grade 2	62 (53.0%)	34 (53.1%)	28 (52.8%)	
Grade 3	22 (18.8%)	11 (17.2%)	11 (20.8%)	
Missing	3	3	0	
Margin				0.069
Free	57 (76.0%)	24 (66.7%)	33 (84.6%)	
Not free	18 (24.0%)	12 (33.3%)	6 (15.4%)	
Missing	45	31	14	
ENE				0.996
No	39 (48.8%)	19 (48.7%)	20 (48.8%)	
Yes	41 (51.2%)	20 (51.3%)	21 (51.2%)	
Missing	40	28	12	
LVI+	88 (73.3%)	50 (74.6%)	38 (71.7%)	0.719
PNI+	32 (26.7%)	17 (25.4%)	15 (28.3%)	0.290
Stage				0.550
Stage I	4 (3.5%)	1 (1.6%)	3 (5.9%)	
Stage II	5 (4.4%)	2 (3.2%)	3 (5.9%)	
Stage III	12 (10.5%)	7 (11.1%)	5 (9.8%)	
Stage IV	99 (82.5%)	57 (85.1%)	42 (79.2%)	
Surgery	84 (70.0%)	41 (61.2%)	43 (81.1%)	**0.018**
Induction	17 (14.2%)	12 (17.9%)	5 (9.4%)	0.186
CCRT response				**<0.001**
CR	55 (45.8%)	41 (61.2%)	14 (26.4%)	
PR	27 (22.5%)	17 (25.4%)	10 (18.9%)	
SD	7 (5.8%)	5 (7.5%)	2 (3.8%)	
PD	31 (25.8%)	4 (6.0%)	27 (50.9%)	
TMB, mut/Mb	3.8 (0.0–25.2)	3.8 (0.0–15.1)	3.8 (0.0–25.2)	0.680
<10	111 (92.5%)	60 (89.6%)	51 (96.2%)	0.296
≥10	9 (7.5%)	7 (10.4%)	2 (3.8%)	
Overall survival				**<0.001**
Died	27 (22.5%)	3 (4.5%)	24 (45.3%)	
Survived	93 (77.5%)	64 (95.5%)	29 (54.7%)	

The *P* value was estimated using the chi-squared test, Fisher’s exact test or Wilcoxon rank sum test.

*p* values that are statistically significant are shown in bold.

Univariate Cox proportional hazard regression analysis was performed to analyze the individual effects of clinicopathological factors and somatic mutation profiles on RFS (Supplementary Table 3). Multiple multivariate Cox regression models were performed (Table 3), and the variance inflation factors (VIFs) of each factor included in the multivariate model were computed to ensure that the included factors had no severe collinearity with each other. First, all somatic mutation profiles were retained in the multivariate model, and clinicopathological factors with *p* < 0.2 in the univariate analysis were included in the multivariate model. Then, the significant factors estimated using the initial multivariate model were abstracted to construct the second model. Furthermore, the significant factors were further mapped to the validation cohort to ensure that the factors included were validated for the validation procedure. The factors missing from the validation cohort were then removed, and the third model was constructed. Next, Harrell’s C-index was computed for each model to identify the best predictive model for relapse risk in the study cohort.

**Fig. 7:**
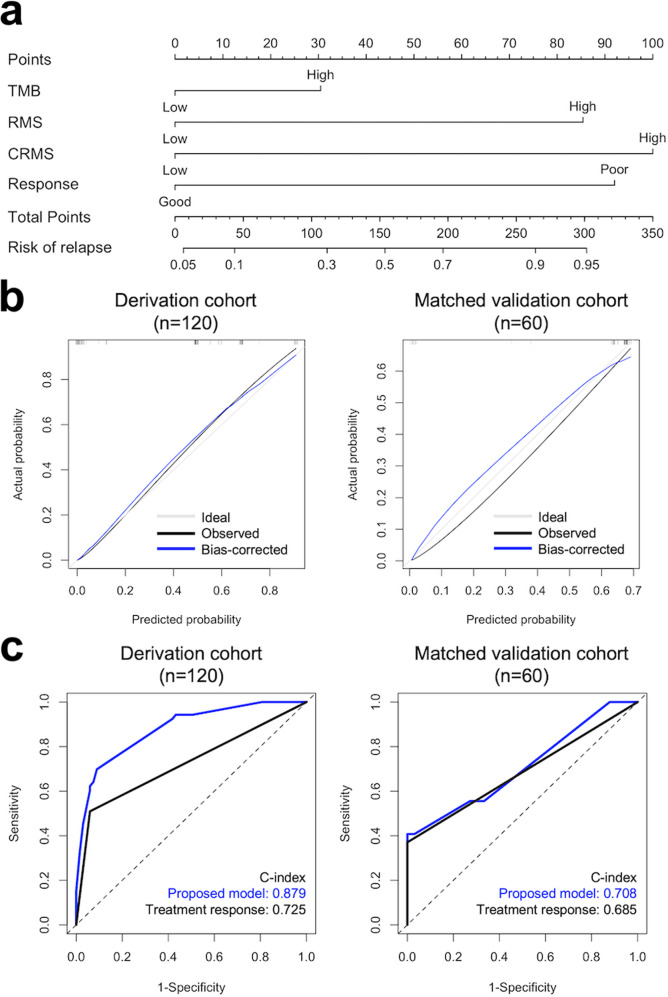
A prognostic nomogram for relapse risk prediction derived from the multivariate model with the best predictive performance. **a** Relapse risk prediction nomogram derived from the multivariate Cox proportional hazard regression model using the derivation cohort. **b** Calibration plot for the nomogram in both the derivation and validation cohorts. **c** Comparison between the predictive performance of the proposed model and treatment response in both cohorts.

**Fig. 8:**
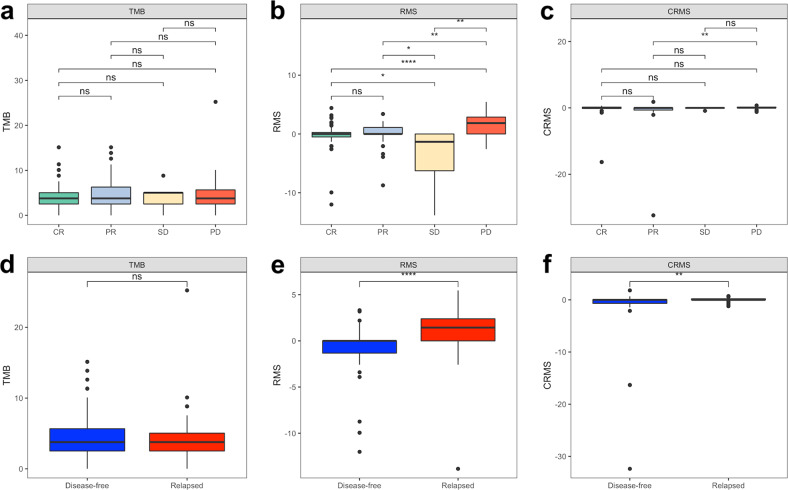
Boxplots of TMB, RMS, and CRMS according to CCRT response and RFS status for the HNSC CCRT cohort. Boxplot of TMB, RMS, and CRMS according to (**a**–**c**) CCRT response and (**d**–**f**) RFS. *p < 0.05, **p < 0.01, ***p < 0.001, ns indicates nonsignificant.

A prognostic nomogram for relapse risk prediction was constructed using significant factors derived from the multivariate model with the best predictive performance (Fig. 7a). Calibration plots for the prognostic nomogram in both the derivation and validation cohorts were illustrated to further validate the model performance (Fig. 7b), and the comparison between the predictive performance of the proposed model and the treatment response in both cohorts was visualized using ROC analysis (Fig. 7c). In addition, TMB, RMS, and CRMS were visualized using boxplots according to CCRT response and RFS status, and pairwise comparisons between subgroups were estimated using the Wilcoxon rank-sum test (Fig. 8). All *p* values were two-sided, and *p* values < 0.05 were considered statistically significant. All analyses were performed using R 4.1.2 (R Core Team, 2022).

## Results

### Patient characteristics

The analysis workflow for the current study is shown in Fig. 1. One hundred and twenty patients with HNSC who had received CCRT were included; all baseline characteristics of our cohort are listed in Table 1. Most patients were between 45 and 64 years old (64.2%), and over 90% of the patients were male. Most patients had oral cavity cancer (65.8%), followed by the oropharynx (20.0%), hypopharynx (10.8%), and larynx (3.3%). Most participants had grade 2 tumors (53.0%), followed by grade 1 (28.2%) and grade 3 (18.8%). Eighty-eight patients had lymphovascular invasion, and 32 patients had perineural invasion. Eighty-four patients underwent surgery and adjuvant CCRT (70%); 19 patients received definitive CCRT, and 17 patients received induction chemotherapy followed by definitive CCRT. Among the patients who underwent surgery, 18 had positive margins, and 41 had ENE. One hundred and eleven patients had locally advanced disease. The mean TMB was 3.8 mut/Mb, and over 90% of the patients had a TMB less than 10 mut/Mb. Of the 120 patients with HNSC, 27 died during the follow-up period, and 93 survived. Fifty-three patients experienced disease relapse after CCRT, and 67 patients did not experience recurrence during the follow-up period. Most baseline characteristics between the relapse and control groups were similar, except for the distribution of surgery, CCRT response, and overall survival. Patients who underwent surgery (*p* = 0.018) had a significantly higher proportion of progressive disease after CCRT (*p* < 0.001), death (*p* < 0.001), and relapse events.

### Landscape of genetic mutation profiles in HNSC

Our 120-patient cohort generated a heatmap of somatic mutation profiles for the top 50 mutated genes according to the targeted sequencing results (Fig. 2). Different mutational types were annotated, including pathogenic, amplification, and promotion. The most frequent mutations in patients with HNSC treated with CCRT were TP53 mutations (81.7%), followed by TERT promoter mutations and CDKN2A mutations (54.2%), NOTCH1 mutations (39.2%), and coamplification of FGF3/FGF4/FGF19 and CCND1 (34.2%). The mutation rates of all detected genes are summarized in Supplementary Table 1. Five of the top 50 mutated genes showed significant RFS differences between mutated and wild types in corresponding genes using the log-rank test. Our results indicated that patients with SOX2 (*p* = 0.007), KLHL6 (*p* = 0.020), and ATR (*p* = 0.044) mutations had significantly better RFS; however, patients with JAK2 (*p* = 0.031) and FANCG (*p* = 0.013) mutations had significantly poorer RFS. Although the significant effects of a single gene could aid in recurrence risk prediction, the cumulative effects between each gene did not increase the significant effects accordingly (Supplementary Fig. 1). In addition, the complex traits of HNSC are still difficult to explain via single-gene effects.

### Relapse mutation signature (RMS) and chromatin remodeling mutation signature (CRMS) of targeted genes

To further decipher the prognostic roles of the genomic mutation signature in HNSC, all patients were divided into wild-type and mutant-type groups according to the gene mutation status of each detected gene, and the individual impact of each gene on RFS was tested using the log-rank test. Thirty candidate genes that were significantly associated with RFS were selected as relapse-associated candidate genes, and the mutation rate of each candidate gene is shown in Fig. 3a. From the NGS gene list, we selected 18 chromatin remodeling-associated genes, 14 of which were mutated in our cohort, including *KMT2D* (30.8%), *CREBBP* (14.2%), *EP300* (10.8%), *SETD2* (10.0%), *TET2* (8.3%), *MLL* (8.3%), *WHSC1L1* (7.5%), *DOT1L* (7.5%), *DNMT3A* (5.0%), *BRD4* (3.3%), *PRDM1* (2.5%), *WHSC1* (1.7%), *HDAC1* (1.7%), and *EZH2* (1.7%). We constructed a chromatin remodeling mutation signature (CRMS) based on these 14 genes (Fig. 3b). Furthermore, we selected a relapse-associated target gene using the LASSO model according to the log lambda value (Fig. 3c, d), and 16 target genes were selected as the optimal combination for the relapse mutation signature (RMS). We generated two multivariate Cox regression models using 16 relapse-associated target genes and 14 chromatin remodeling-associated genes (Table 2). A higher coefficient indicates that the corresponding gene mutation obtained a higher risk of RFS, and a lower coefficient indicates that the corresponding gene mutation obtained a lower risk of RFS. Although some genes showed relatively superior coefficients, a large standard error (SE) indicates a large discrepancy among sample distributions. Hence, the coefficient results derived by multivariate Cox regression for each gene set might not be appropriate when directly used to interpret the impact of gene mutation on RFS. Therefore, the coefficient of each gene was then abstracted to generate RMS and CRMS to represent the somatic mutation signatures of the study cohort. Moreover, the OncoPrint diagram of mutational frequencies and alterations of the 16 RMS genes and 14 CRMS genes sequenced are shown in Fig. 4a, b, respectively.

**Table 2:** Multivariate Cox regression analysis for 16 target genes selected by LASSO coefficient profiles according to relapse-free survival (RFS) and 14 chromatic remodeling-associated genes.

No	RMS genes	Coefficients (β)	SE	*P*	CRMS genes	Coefficients (β)	SE	*P*
1	SOX2	−2.068	0.552	**<0.001**	MLL	−0.341	0.693	0.622
2	ATR	−0.510	0.602	0.397	SETD2	0.466	0.432	0.281
3	JAK2	1.975	0.432	**<0.001**	EP300	−0.884	0.636	0.165
4	FANCG	1.436	0.427	**<0.001**	KMT2D	0.213	0.331	0.520
5	ZNF217	1.843	0.415	**<0.001**	BRD4	0.021	1.054	0.984
6	EPHB4	−1.330	1.018	0.191	CREBBP	0.051	0.442	0.908
7	MUTYH	1.204	0.493	**0.015**	DOT1L	0.484	0.479	0.311
8	AXL	0.367	0.481	0.445	TET2	−0.295	0.626	0.637
9	CD22	−9.944	115.986	0.932	PRDM1	0.278	1.203	0.817
10	CDKN1A	2.836	0.613	**<0.001**	WHSC1L1	−1.436	1.025	0.161
11	ERBB3	1.745	0.611	**0.004**	DNMT3A	−0.186	0.597	0.755
12	RAD51D	1.740	0.613	**0.005**	WHSC1	−16.337	4702.509	0.997
13	STAT3	2.194	0.623	**<0.001**	HDAC1	−16.330	4745.240	0.997
14	ABL1	1.365	0.621	**0.028**	EZH2	1.581	1.424	0.267
15	MITF	2.574	0.741	**<0.001**	–			
16	PAX5	1.003	0.807	0.214	–			

*HR* hazard ratio, *SE* standard error.

*p* values that are statistically significant are shown in bold.

### Prognostic impact of TMB, RMS, and CRMS in patients with HNSC who underwent CCRT

The correlation between TMB, RMS, and CRMS is illustrated in Fig. 5a, where RMS and CRMS showed a significantly weak positive correlation with each other. However, TMB showed no significant correlation with RMS or CRMS. Furthermore, we evaluated the predictive performance of TMB, RMS, and CRMS with respect to RFS using ROC analysis. Individual continuous RMS (AUC = 0.746) showed better predictive performance than continuous CRMS (AUC = 0.657) and TMB (AUC = 0.522), as shown in Fig. 5b, c. The addition of continuous TMB only improved the performance of continuous CRMS (CRMS + TMB, AUC = 0.681; Fig. 5c). Moreover, the addition of RMS and CRMS resulted in a superior performance increase (CRMS + RMS, AUC = 0.784; Fig. 5d). The optimal cutoff values of TMB, RMS and CRMS were chosen according to the ROC analysis results. First, the discriminant performance of each value within TMB, RMS and CRMS was determined and reported using the AUC value, and a higher AUC indicated better predictive performance. Afterward, the corresponding value of TMB, RMS and CRMS that obtained the highest AUC was selected as an optimal cutoff value. The optimal cutoff values of TMB, RMS, and CRMS were 3.78, 1.135, and −0.62, respectively. For instance, patients with a TMB ≥ 3.78 might have greater recurrence risk discrepancy than those with a TMB < 3.78. Consistently, patients with RMS ≥ 1.135 and CRMS ≥ −0.62 might also have greater risk discrepancy compared to those with RMS < 1.135 and CRMS < −0.62. Based on the optimal cutoff values of TMB, RMS, and CRMS, all patients were categorized into high and low categories, and survival differences according to the individual and pairwise combinations of TMB, RMS, and CRMS subgroups were estimated and are summarized in Fig. 6. Although poor survival trends were observed in the high-TMB, high-RMS, and high-CRMS subgroups, RFS analysis results showed significantly poor RFS in the individual RMS (*p* < 0.001; Fig. 6b) and CRMS (*p* = 0.006; Fig. 6c) subgroups only.

To improve the predictive ability, pairwise combinations, including RMS-TMB, CRMS-TMB, and RMS-CRMS subgroups, were analyzed. In the RMS-TMB subgroup, the both-high group demonstrated a higher relapse risk than the both-low group (*p* < 0.001; Fig. 6d). In the CRMS-TMB subgroup, the both-high group demonstrated a higher risk of relapse than the both-low group (*p* = 0.020; Fig. 6e). In the RMS-CRMS subgroup, the both-high group demonstrated a higher risk of relapse than patients in the both-low group (*p* < 0.001; Fig. 6f). Our results revealed that the pairwise combination evaluation of these three biomarkers and signatures could improve the efficiency of prognostic risk prediction. Notably, the high-RMS and high-CRMS subgroups showed consistently poor RFS in pairwise combination analysis (Fig. 6d–f). The clinical and sequencing data retrieved from TCGA-HNSC projects were used to further validate the performance of RMS and CRMS. A total of 160 HNSC patients who had ever received CCRT were enrolled, and the validation results are summarized in Supplementary Table 2 and Supplementary Fig. 2. According to Supplementary Table 1, the most frequently somatic mutated RMS- and CRMS-related genes in the overall validation cohort were KMT2D (16.9%), CREBBP (8.1%), EP300 (7.5%), ATR (6.2%), and DOT1L (5.0%). However, in the Asian cohort, only SOX2, EPHB4, EP300, KMT2D, DOT1L, and EZH2 gene mutations were observed. Mutations in three genes each in RMS (FANCG, AXL, and CDKN1A) and CRMS (MLL, PRDM1, and HDAC1) were not observed in the validation cohort. Considering the heterogeneity of ethnicity, we performed validation in the overall cohort and in the Asian and non-Asian cohorts. As shown in Supplementary Fig. 2, the prognostic risk predictive ability, satisfactory predictive performance, and boxplot of RMS and CRMS showed better results in the Asian cohort (Supplementary Fig. 2b) than in the overall cohort (Supplementary Fig. 2a) and the non-Asian cohort (Supplementary Fig. 2c). The validation results indicate that the prognostic risk predictive ability of RMS and CRMS contributed more to the Asian HNSC cohort.

### Establishment and assessment of the nomogram

To identify independent prognostic and predictive biomarkers, we used univariate Cox regression analysis to evaluate the individual association between RFS and clinicopathological factors (Supplementary Table 3). All somatic mutation profiles were retained, and the clinicopathological factors with *p* < 0.2 in the univariate analysis, including location, margin, ENE, PNI, and CCRT response, were included in the multivariate Cox regression model. Multivariate Cox regression analysis showed that ENE, CCRT response, and the three somatic mutation profiles were significant predictors of RFS in the HNSC CCRT cohort. As mentioned before, a higher TMB value (≥3.78 mut/Mb) demonstrated a significant predictive effect for RFS than a lower TMB value after multivariate Cox regression analysis (*p* = 0.017). A higher RMS level (≥1.135) was a more powerful predictor of shorter RFS after adjusting for other clinical characteristics (*p* < 0.001). Similarly, a higher CRMS level (≥−0.62) was also significantly predictive of early cancer relapse in the multivariate analysis (*p* = 0.005). The comprehensive univariate Cox regression analysis results of the derivation cohort are summarized in Supplementary Table 3. All multivariate Cox regression analysis results of the derivation cohort are presented in Table 3. Model A abstracted the multivariate estimation results of significant factors for relapse risk according to Supplementary Table 3. Model B included only the factors that were significant in Model A. While mapping to the validation cohort, ENE status was unable to be collected due to the retrospective nature of the TCGA-HNSC database. Therefore, we removed ENE status and constructed Model C. Overall, Model C had the highest C-index with 0.879 (95% CI = 0.821–0.938), indicating better prediction performance for relapse risk compared to Model A (C-index = 0.836, 95% CI = 0.763–0.909) and Model B (C-index = 0.873, 95% CI = 0.811–0.934). Therefore, the factors included in Model C were used to construct the relapse risk prediction nomogram as shown in Fig. 7a, including treatment response and somatic mutation profiles.

**Table 3:** Multivariate cox regression analysis results of the derivation cohort.

Factors	Comparison	Model A^a^		Model B^b^		Model C^c^	
		HR (95% CI)	*P*	HR (95% CI)	*P*	HR (95% CI)	*P*
*Clinicopathological factors*							
ENE	Yes vs. No	3.23 (1.55, 6.70)	0.002	2.35 (1.28, 4.32)	0.006	–	
Response^d^	Poor vs. Good	6.7 (3.30, 13.6)	<0.001	7.36 (3.85, 14.1)	<0.001	6.26 (3.33, 11.8)	<0.001
*Somatic mutation profiles*							
TMB, mut/Mb	High (≥3.78) vs. Low	2.29 (1.16, 4.53)	0.017	1.84 (0.98, 3.43)	0.056	1.84 (0.98, 3.44)	0.058
RMS	High (≥1.135) vs. Low	5.73 (2.61, 12.6)	<0.001	5.87 (2.99, 11.5)	<0.001	5.49 (2.79, 10.8)	<0.001
CRMS	High (≥−0.62) vs. Low	8.42 (1.77, 40.1)	0.007	6.13 (1.43, 26.3)	0.015	7.34 (1.73, 31.1)	0.007
*Harrel’s C-index*^*e*^		0.836 (0.763–0.909)		0.873 (0.811–0.934)		0.879 (0.821–0.938)	

^a^Model A abstracted the multivariate estimation results of significant factors for relapse risk according to Supplementary Table 3. All somatic mutation profiles were retained in the multivariate model, and clinicopathological factors with *p* < 0.2 in univariate analysis results were included in the multivariate model.

^b^Model B included only the factors that were significant in Model A.

^c^Model C included CCRT response and somatic mutation profiles only because the ENE status was lacking in the validation cohort.

^d^Poor response indicates patients with PD response after receiving CCRT, and good response indicates patients with either CR, PR or SD response after receiving CCRT.

^e^Harrell’s C-index was computed to estimate the model performance between three different multivariate models, and the model with the highest C-index indicated better predictive performance.

As described previously, the TCGA-HNSC validation cohort was mostly non-Asian. Therefore, we matched the TCGA-HNSC database using age group, sex and treatment response proportion of our study cohort to reduce the discrepancy between the derivation and validation cohorts. In summary, our proposed risk predictive model was generated using a derivation cohort of 120 HNSC patients and validated using a matched validation cohort of 60 patients abstracted from the TCGA-HNSC database. In addition, the clinicopathological factors and somatic mutation profiles of the validation cohort are summarized in Supplementary Table 4. Figure 7b demonstrates the calibration plots for the prognostic nomogram in both the derivation and validation cohorts, and the calibration results revealed good agreement between the model-predicted and actual observed relapse risk survival outcomes in the derivation cohort. The agreement for the matched validation cohort was decreased but still acceptable and close to the ideal curve. In addition, the predictive performance of the proposed model and the treatment response in both cohorts were visualized using ROC analysis, as shown in Fig. 7c. The comparison results showed that somatic mutation profiles could improve the relapse risk predictive performance of treatment response in both cohorts, and the C-index of the proposed model showed good predictive ability for the derivation (C-index = 0.879) and matched validation cohorts (C-index = 0.708).

To clarify the relationship between somatic mutation profiles, CCRT response, and RFS status, we observed the distribution of TMB, RMS, and CRMS among different characteristics (Fig. 8). Figure 8a shows no significant difference in TMB between different CCRT responses. Figure 8b shows a markedly significant difference in RMS between the CR and PD groups (*p* < 0.001). Figure 8c also shows a significant difference in CRMS between the PR and PD groups (*p* < 0.010). Then, we compared TMB with relapse status, and there was still no significant difference between the disease-free and relapse groups (Fig. 8d). Figure 8e shows that RMS in the relapsed group was significantly higher than that in the disease-free group (*p* < 0.001). CRMS was also consistently significantly higher in the relapse group than in the disease-free group (*p* < 0.010) (Fig. 8f).

Collectively, we discovered two gene signatures associated with disease relapse and chromatin remodeling, RMS and CRMS, that can precisely predict the treatment response and cancer recurrence. To reinforce the efficiency of genetic testing, we organized the clinical characteristics and genetic testing and established a comprehensive nomogram to assess the relapse risk of the HNSC post-CCRT cohort.

## Discussion

In this study, we explored the role of genetic mutational signatures and TMB in the clinical prognosis of HNSC. First, NGS with targeted sequencing data for patients with HNSC treated with CCRT was obtained. Second, we performed the somatic mutation rate and RFS analysis using the log-rank test for all detected genes in the study cohort. Third, LASSO, univariate, and multivariate Cox regression analyses were used to screen for possible prognostic mutational gene signatures regarding both relapse outcome and chromatin remodeling, which are frequently associated with DDR, and then a mutation-risk model was constructed for predicting disease recurrence. After confirming the predictive role of RMS and CRMS, we incorporated TMB values and clinical characteristics and constructed a nomogram for further assessment. RMS and CRMS were confirmed to be independent predictors of CCRT response and RFS in patients with HNSC post-CCRT.

Carcinogen exposure, which is the most potent risk factor for non-HPV HNSC formation, leads to genetic alterations and then to dysregulation of metabolism^21^. According to The Cancer Genome Atlas (TCGA) data, most smoking-related HNSCs demonstrate loss-of-function *TP53* mutations and *CDKN2A* inactivation. HPV-associated HNSC is mainly related to helical domain mutations in *PIK3CA*, loss of *TRAF3*, and amplification of *E2F1*^22–24^. In our study, the most relevant mutation was *TP53* (81.7%), followed by the *TERT* promoter (54.2%), *CDKN2A* (54.2%), *NOTCH1* (39.2%), and coamplification of *FGF3/FGF4/FGF19* and *CCND1* (34.2%). Compared with previous studies that used whole-exome sequencing, a higher incidence of *TERT* promoter mutations and *FGF*-associated mutations was found in our study. A recent study by Moreira et al. used an in-house targeted NGS panel (571 genes) to detect the targeted genes in 151 patients with surgical HNSC and was more consistent with our study, except for a higher incidence of *FGF*-associated mutations in our cohort. According to their study, *TP53* was the most mutated gene (71%), followed by alterations in the *TERT* promoter (50%), *CDKN2A* (25%), *FAT1* (17%), *PIK3CA* (14%), and *NOTCH1* (15%).

Epigenetic mechanisms regulating genomic structure and function play important roles in head and neck carcinogenesis^25,26^. They are involved in multiple levels of gene regulation, including DNA methylation, chromatin remodeling, histone posttranslational covalent modifications, and the effects of noncoding RNA. In our study, *KMT2D*, a histone methyltransferase harboring somatic mutations, was the most frequent chromatin remodeling mutated gene (30.8%), which was higher than in the TCGA data (~17%)^22^. *CREBBP* and *EP300*, mutated in 13% of HNSC cases, are associated with synthetic cytotoxicity and recurrence following radiation^27^, which is consistent with our study. *SETD2* and *TET2* were altered in 3.72% and 3.46% of patients with HNSC, respectively, as reported by the AACR Project GENIE^28^. In our study, the incidence of *SETD2* and *TET2* mutations was relatively higher (10.0% and 8.3%, respectively); however, SETD2 was associated with a higher relapse rate, and TET2 was associated with a lower relapse rate. Compared with the dataset of 300 patients with HNSC in the AACR Project GENIE, our cohort was relatively small and recruited only Taiwanese patients. These factors may have caused our results to differ from those of other databases. However, we investigated the possible relationship between epigenetic alterations and patient prognosis and developed a risk predictive signature to correlate with CCRT response and RFS. CRMS, composed of 14 chromatin remodeling mutated genes, is a reliable biomarker for predicting disease relapse and can be applied in clinical practice.

Recently, immune checkpoint inhibitors (ICIs) have become promising agents for treating HNSC. Monoclonal antibodies targeting anti-programmed death protein-1 (anti–PD-1), nivolumab and pembrolizumab, demonstrated stunning and durable therapeutic efficacy in HNSC^29,30^. However, the available clinical biomarkers associated with PD-L1, including tumor cell and immune cell expression, are not reliable in predicting responses to immunotherapy^31,32^. TMB represents the number of mutations per megabase (mut/Mb) of DNA in specific cancers and was initially identified as a biomarker for ICIs in melanoma and subsequently explored in lung cancer^33^. An increased TMB level was regarded as a higher response to immunotherapy due to increased neoantigen formation and immune activation. There is still no definite consensus on TMB thresholds, although some studies have reported that TMB thresholds proposed ~200 nonsynonymous somatic mutations by WES^34^. In a recent study, higher TMB values (the highest 20% TMB values, 11.5 mut/Mb) demonstrated a more favorable prognosis in patients with oral cancer treated with upfront surgery^15^. In our study, a higher TMB value (cutoff 3.78 mut/Mb) demonstrated a significant predictive effect for RFS in patients treated with CCRT than in those with a lower TMB value in the multivariate analysis. In addition, over 90% of the patients in our cohort had TMB values <10 mut/Mb. Notably, the TMB values of their cohort were higher than those of our cohort, and this difference was due to the difference in the TMB calculation. From the TCGA database, higher TMB determined by WES analysis was associated with shorter OS in patients with all stages of HNSC^35^. In accordance with previous findings, including different tumor types, our report suggests that increased TMB is associated with a poor prognosis. For its impact on the clinical outcomes, we incorporated the TMB value into our nomogram to enhance predictive efficiency and risk evaluation.

In previous studies, there were associations between DNA damage and gene listing in RMS and CRMS. Downregulation of *EZH2* (polycomb protein histone methyltransferase enhancer of Zeste homolog 2) rapidly elicits DNA damage and induces p21 (*CDKN1A*) expression, triggering cell senescence^36^. In glioblastoma multiforme (GBM), which is often treated with concurrent chemoradiotherapy with temozolomide, *EZH2* binds to and methylates *STAT3*, leading to enhanced *STAT3* activity by phosphorylation of *STAT3* and promoting the tumorigenicity of GBM stem-like cells^37^. In nasopharyngeal carcinoma cells (NPCs), β-elemene inhibits DNA damage repair and NPC cell growth via inactivation of *Stat3* and reduces *DNMT1* and *EZH2* expression. The interplay of *DNMT1* and *EZH2* and the mutual regulation of *Stat3*, *EZH2* and *DNMT1* contribute to therapeutic efficacy^38,39^. Furthermore, p21 reduced transcriptional activation by STAT3 proteins and interacted with the CREB-binding coactivator protein. Interestingly, the inhibitory effect of STAT3 on p21 was not observed when the CREB-binding protein was overexpressed^40^. According to previous studies, the p21-STAT3 interaction could potentially affect/coregulate gene expression in DNA damage and cell cycle arrest^40,41^, which is consistent with the findings of the current study. CRMS (DNA repair-associated) and RMS (p21-STAT3 interaction) interaction terms also showed a potential add-on value in prognostic risk prediction in the study cohort.

Our study had several limitations. First, the primary sites of our cohort included the oral cavity, oropharynx, hypopharynx, and larynx, which may interfere with the results due to the heterogeneity of molecular subtypes from different anatomic sites. Second, the therapeutic interventions for all patients were not consistent due to multimodality management, including induction chemotherapy, upfront surgery, radiotherapy alone, and definite and adjuvant CCRT. Third, the analysis of our study was restricted to targeted sequences by using NGS assays, not whole-exome sequences. Some genetic alterations involving somatic point mutations as well as many types of structural variants were not thoroughly explored in our report. Finally, our analysis was restricted to genetic alterations, and proteomic and phenotypic alterations were lacking. Thus, larger cohorts are warranted for further comprehensive evaluation. Different integrated parameters, including clinical, pathological, and genetic profiles, were used to meditate and compensate for the insufficiencies of our analysis. Although the validation results showed the potential prognostic risk predictive ability of RMS and CRMS, the limited Asian HNSC patients restricted further investigation, including Cox model estimation or nomogram prediction. Nevertheless, this study still provides valuable information for genomic investigation of the Asian HNSC population.

In conclusion, this study provides an integrative analysis of the clinical parameters, pathological characteristics, and genetic profiles of patients with HNSC treated with CCRT. To explore the depth of genomic analysis, we incorporated tumor mutational burden and relapse-associated and epigenetic-associated mutated gene signatures to design a comprehensive nomogram for evaluating cancer relapse in HNSC. Our findings indicated that clinical or histological risk evaluation was not sufficient for the prediction of HNSC, and an in-depth interpretation of genetic profiling may be a mandatory process in the future.

## Supplementary information

Supplementary data

## Data Availability

The datasets presented in this paper are not readily available because of patient confidentiality and participant privacy terms. Requests to access the datasets should be directed to the corresponding author on reasonable request.
