# Supplementary material for: The role of the genomic mutation signature and tumor mutation burden on relapse risk prediction in head and neck squamous cell carcinoma after concurrent chemoradiotherapy
Source: Exp Mol Med. 2023 May 1;55(5):926–38. doi: 10.1038/s12276-023-00984-4 (PMC10238468; doi:10.1038/s12276-023-00984-4)

Supplementary Table 1. Individual somatic mutation rate and survival analysis results for each detected gene in the study cohort.

Gene	Overall (n=120)	Disease-free (n=67)	Relapsed (n=53)	*P* (log-rank test)
**TP53**	98 (81.7%)	54 (80.6%)	44 (83.0%)	0.991
**CDKN2A**	65 (54.2%)	36 (53.7%)	29 (54.7%)	0.658
**TERT**	65 (54.2%)	32 (47.8%)	33 (62.3%)	0.076
**NOTCH1**	47 (39.2%)	24 (35.8%)	23 (43.4%)	0.171
**FGF19**	41 (34.2%)	22 (32.8%)	19 (35.8%)	0.337
**FGF3**	41 (34.2%)	23 (34.3%)	18 (34.0%)	0.619
**FGF4**	39 (32.5%)	20 (29.9%)	19 (35.8%)	0.217
**CCND1**	38 (31.7%)	20 (29.9%)	18 (34.0%)	0.168
**KMT2D**	37 (30.8%)	21 (31.3%)	16 (30.2%)	0.577
**PIK3CA**	29 (24.2%)	16 (23.9%)	13 (24.5%)	0.378
**CASP8**	24 (20.0%)	12 (17.9%)	12 (22.6%)	0.548
**PRKCI**	22 (18.3%)	14 (20.9%)	8 (15.1%)	0.056
**SOX2**	22 (18.3%)	15 (22.4%)	7 (13.2%)	0.007
**CDKN2B**	21 (17.5%)	14 (20.9%)	7 (13.2%)	0.124
**KLHL6**	19 (15.8%)	13 (19.4%)	6 (11.3%)	0.020
**ARID1A**	18 (15.0%)	12 (17.9%)	6 (11.3%)	0.575
**EGFR**	18 (15.0%)	8 (11.9%)	10 (18.9%)	0.114
**CREBBP**	17 (14.2%)	10 (14.9%)	7 (13.2%)	0.817
**PIK3C2G**	17 (14.2%)	11 (16.4%)	6 (11.3%)	0.549
**TIPARP**	17 (14.2%)	12 (17.9%)	5 (9.4%)	0.117
**ATM**	16 (13.3%)	9 (13.4%)	7 (13.2%)	0.739
**BCL6**	15 (12.5%)	8 (11.9%)	7 (13.2%)	0.198
**MAP3K13**	15 (12.5%)	8 (11.9%)	7 (13.2%)	0.089
**TERC**	15 (12.5%)	10 (14.9%)	5 (9.4%)	0.142
**ATR**	14 (11.7%)	10 (14.9%)	4 (7.5%)	0.044
**FGF12**	14 (11.7%)	11 (16.4%)	3 (5.7%)	0.069
**MSH6**	14 (11.7%)	7 (10.4%)	7 (13.2%)	0.715
**ROS1**	14 (11.7%)	9 (13.4%)	5 (9.4%)	0.728
**ALK**	13 (10.8%)	5 (7.5%)	8 (15.1%)	0.336
**BRCA2**	13 (10.8%)	6 (9.0%)	7 (13.2%)	0.994
**EP300**	13 (10.8%)	9 (13.4%)	4 (7.5%)	0.497
**NOTCH3**	13 (10.8%)	7 (10.4%)	6 (11.3%)	0.755
**PTCH1**	13 (10.8%)	7 (10.4%)	6 (11.3%)	0.550
**ASXL1**	12 (10.0%)	8 (11.9%)	4 (7.5%)	0.516
**BRIP1**	12 (10.0%)	6 (9.0%)	6 (11.3%)	0.320
**JAK2**	12 (10.0%)	5 (7.5%)	7 (13.2%)	0.031

**MYC**	12 (10.0%)	8 (11.9%)	4 (7.5%)	0.952
**SDHA**	12 (10.0%)	8 (11.9%)	4 (7.5%)	0.607
**SETD2**	12 (10.0%)	4 (6.0%)	8 (15.1%)	0.262
**SPEN**	12 (10.0%)	6 (9.0%)	6 (11.3%)	0.421
**CARD11**	11 (9.2%)	9 (13.4%)	2 (3.8%)	0.335
**ERBB2**	11 (9.2%)	8 (11.9%)	3 (5.7%)	0.631
**MSH3**	11 (9.2%)	9 (13.4%)	2 (3.8%)	0.392
**NOTCH2**	11 (9.2%)	7 (10.4%)	4 (7.5%)	0.548
**PTEN**	11 (9.2%)	7 (10.4%)	4 (7.5%)	0.845
**FANCG**	10 (8.3%)	3 (4.5%)	7 (13.2%)	0.013
**HRAS**	10 (8.3%)	7 (10.4%)	3 (5.7%)	0.436
**KDM5A**	10 (8.3%)	3 (4.5%)	7 (13.2%)	0.637
**MLL**	10 (8.3%)	5 (7.5%)	5 (9.4%)	0.950
**MTAP**	10 (8.3%)	6 (9.0%)	4 (7.5%)	0.715
**TET2**	10 (8.3%)	7 (10.4%)	3 (5.7%)	0.488
**DOT1L**	9 (7.5%)	3 (4.5%)	6 (11.3%)	0.395
**ERBB4**	9 (7.5%)	5 (7.5%)	4 (7.5%)	0.813
**FANCC**	9 (7.5%)	6 (9.0%)	3 (5.7%)	0.520
**GNAS**	9 (7.5%)	4 (6.0%)	5 (9.4%)	0.413
**NFE2L2**	9 (7.5%)	6 (9.0%)	3 (5.7%)	0.527
**PDCD1LG2**	9 (7.5%)	6 (9.0%)	3 (5.7%)	0.265
**PTPRO**	9 (7.5%)	6 (9.0%)	3 (5.7%)	0.151
**RAD21**	9 (7.5%)	4 (6.0%)	5 (9.4%)	0.394
**TSC1**	9 (7.5%)	3 (4.5%)	6 (11.3%)	0.187
**TSC2**	9 (7.5%)	6 (9.0%)	3 (5.7%)	0.462
**WHSC1L1**	9 (7.5%)	8 (11.9%)	1 (1.9%)	0.166
**ZNF217**	9 (7.5%)	2 (3.0%)	7 (13.2%)	0.001
**ZNF703**	9 (7.5%)	5 (7.5%)	4 (7.5%)	0.608
**BCORL1**	8 (6.7%)	5 (7.5%)	3 (5.7%)	0.723
**EPHB4**	8 (6.7%)	6 (9.0%)	2 (3.8%)	0.050
**ERCC4**	8 (6.7%)	4 (6.0%)	4 (7.5%)	0.647
**FANCA**	8 (6.7%)	5 (7.5%)	3 (5.7%)	0.326
**FGF10**	8 (6.7%)	7 (10.4%)	1 (1.9%)	0.114
**GRM3**	8 (6.7%)	5 (7.5%)	3 (5.7%)	0.896
**KDR**	8 (6.7%)	4 (6.0%)	4 (7.5%)	0.310
**KIT**	8 (6.7%)	5 (7.5%)	3 (5.7%)	0.265
**LTK**	8 (6.7%)	6 (9.0%)	2 (3.8%)	0.669
**MERTK**	8 (6.7%)	3 (4.5%)	5 (9.4%)	0.781
**MTOR**	8 (6.7%)	6 (9.0%)	2 (3.8%)	0.411
**MUTYH**	8 (6.7%)	3 (4.5%)	5 (9.4%)	0.010
**NTRK3**	8 (6.7%)	7 (10.4%)	1 (1.9%)	0.220

**PARP3**	8 (6.7%)	4 (6.0%)	4 (7.5%)	0.003
**PMS2**	8 (6.7%)	3 (4.5%)	5 (9.4%)	0.557
**POLE**	8 (6.7%)	7 (10.4%)	1 (1.9%)	0.123
**RET**	8 (6.7%)	6 (9.0%)	2 (3.8%)	0.994
**RICTOR**	8 (6.7%)	6 (9.0%)	2 (3.8%)	0.255
**STK11**	8 (6.7%)	6 (9.0%)	2 (3.8%)	0.416
**CBL**	7 (5.8%)	4 (6.0%)	3 (5.7%)	0.619
**CDKN1B**	7 (5.8%)	4 (6.0%)	3 (5.7%)	0.889
**EMSY**	7 (5.8%)	4 (6.0%)	3 (5.7%)	0.472
**IGF1R**	7 (5.8%)	5 (7.5%)	2 (3.8%)	0.368
**IRS2**	7 (5.8%)	6 (9.0%)	1 (1.9%)	0.162
**JAK3**	7 (5.8%)	2 (3.0%)	5 (9.4%)	0.078
**KEL**	7 (5.8%)	4 (6.0%)	3 (5.7%)	0.181
**MST1R**	7 (5.8%)	3 (4.5%)	4 (7.5%)	0.930
**MYCN**	7 (5.8%)	4 (6.0%)	3 (5.7%)	0.133
**NTRK1**	7 (5.8%)	4 (6.0%)	3 (5.7%)	0.636
**PIK3CB**	7 (5.8%)	4 (6.0%)	3 (5.7%)	0.346
**POLD1**	7 (5.8%)	5 (7.5%)	2 (3.8%)	0.272
**TNFAIP3**	7 (5.8%)	4 (6.0%)	3 (5.7%)	0.871
**APC**	6 (5.0%)	5 (7.5%)	1 (1.9%)	0.369
**AXL**	6 (5.0%)	1 (1.5%)	5 (9.4%)	0.040
**CD274**	6 (5.0%)	3 (4.5%)	3 (5.7%)	0.013
**CIC**	6 (5.0%)	4 (6.0%)	2 (3.8%)	0.627
**DIS3**	6 (5.0%)	6 (9.0%)	0 (0.0%)	0.116
**DNMT3A**	6 (5.0%)	1 (1.5%)	5 (9.4%)	0.936
**EPHB1**	6 (5.0%)	3 (4.5%)	3 (5.7%)	0.293
**FAS**	6 (5.0%)	5 (7.5%)	1 (1.9%)	0.296
**FLT3**	6 (5.0%)	5 (7.5%)	1 (1.9%)	0.193
**KRAS**	6 (5.0%)	4 (6.0%)	2 (3.8%)	0.197
**MAP2K2**	6 (5.0%)	3 (4.5%)	3 (5.7%)	0.176
**MAP3K1**	6 (5.0%)	3 (4.5%)	3 (5.7%)	0.481
**MDM2**	6 (5.0%)	3 (4.5%)	3 (5.7%)	0.452
**NBN**	6 (5.0%)	2 (3.0%)	4 (7.5%)	0.622
**NFKBIA**	6 (5.0%)	3 (4.5%)	3 (5.7%)	0.195
**PALB2**	6 (5.0%)	5 (7.5%)	1 (1.9%)	0.254
**TBX3**	6 (5.0%)	4 (6.0%)	2 (3.8%)	0.952
**TGFBR2**	6 (5.0%)	2 (3.0%)	4 (7.5%)	0.109
**ACVR1B**	5 (4.2%)	4 (6.0%)	1 (1.9%)	0.700
**ARAF**	5 (4.2%)	3 (4.5%)	2 (3.8%)	0.687
**AXIN1**	5 (4.2%)	3 (4.5%)	2 (3.8%)	0.743
**BRAF**	5 (4.2%)	3 (4.5%)	2 (3.8%)	0.318

**BRCA1**	5 (4.2%)	3 (4.5%)	2 (3.8%)	0.607
**CD22**	5 (4.2%)	4 (6.0%)	1 (1.9%)	0.043
**CDK12**	5 (4.2%)	5 (7.5%)	0 (0.0%)	0.117
**CDK6**	5 (4.2%)	5 (7.5%)	0 (0.0%)	0.132
**CHEK1**	5 (4.2%)	3 (4.5%)	2 (3.8%)	0.402
**CRKL**	5 (4.2%)	2 (3.0%)	3 (5.7%)	0.006
**CSF1R**	5 (4.2%)	2 (3.0%)	3 (5.7%)	0.251
**CTCF**	5 (4.2%)	3 (4.5%)	2 (3.8%)	0.849
**EPHA3**	5 (4.2%)	1 (1.5%)	4 (7.5%)	0.074
**FGFR1**	5 (4.2%)	3 (4.5%)	2 (3.8%)	0.330
**FLT1**	5 (4.2%)	2 (3.0%)	3 (5.7%)	0.798
**LYN**	5 (4.2%)	3 (4.5%)	2 (3.8%)	0.638
**MED12**	5 (4.2%)	5 (7.5%)	0 (0.0%)	0.126
**MET**	5 (4.2%)	1 (1.5%)	4 (7.5%)	0.485
**NF1**	5 (4.2%)	5 (7.5%)	0 (0.0%)	0.188
**NKX2.1**	5 (4.2%)	5 (7.5%)	0 (0.0%)	0.235
**PARP1**	5 (4.2%)	4 (6.0%)	1 (1.9%)	0.542
**PARP2**	5 (4.2%)	2 (3.0%)	3 (5.7%)	0.926
**PDGFRA**	5 (4.2%)	2 (3.0%)	3 (5.7%)	0.438
**RB1**	5 (4.2%)	4 (6.0%)	1 (1.9%)	0.263
**RNF43**	5 (4.2%)	3 (4.5%)	2 (3.8%)	0.345
**RPTOR**	5 (4.2%)	3 (4.5%)	2 (3.8%)	0.825
**SGK1**	5 (4.2%)	3 (4.5%)	2 (3.8%)	0.991
**SMARCA4**	5 (4.2%)	3 (4.5%)	2 (3.8%)	0.721
**ATRX**	4 (3.3%)	1 (1.5%)	3 (5.7%)	0.928
**BAP1**	4 (3.3%)	2 (3.0%)	2 (3.8%)	0.927
**BCL2L1**	4 (3.3%)	3 (4.5%)	1 (1.9%)	0.324
**BRD4**	4 (3.3%)	3 (4.5%)	1 (1.9%)	0.764
**CALR**	4 (3.3%)	2 (3.0%)	2 (3.8%)	0.695
**CDKN1A**	4 (3.3%)	1 (1.5%)	3 (5.7%)	0.012
**ERBB3**	4 (3.3%)	1 (1.5%)	3 (5.7%)	0.016
**FBXW7**	4 (3.3%)	3 (4.5%)	1 (1.9%)	0.684
**FGF6**	4 (3.3%)	2 (3.0%)	2 (3.8%)	0.343
**FGFR3**	4 (3.3%)	1 (1.5%)	3 (5.7%)	0.570
**FLCN**	4 (3.3%)	3 (4.5%)	1 (1.9%)	0.236
**GID4**	4 (3.3%)	3 (4.5%)	1 (1.9%)	0.996
**HSD3B1**	4 (3.3%)	3 (4.5%)	1 (1.9%)	0.682
**INPP4B**	4 (3.3%)	3 (4.5%)	1 (1.9%)	0.412
**KDM6A**	4 (3.3%)	2 (3.0%)	2 (3.8%)	0.662
**MAF**	4 (3.3%)	1 (1.5%)	3 (5.7%)	0.321
**MSH2**	4 (3.3%)	3 (4.5%)	1 (1.9%)	0.448

**PDK1**	4 (3.3%)	2 (3.0%)	2 (3.8%)	0.792
**RAD51D**	4 (3.3%)	1 (1.5%)	3 (5.7%)	0.005
**SNCAIP**	4 (3.3%)	1 (1.5%)	3 (5.7%)	0.065
**STAT3**	4 (3.3%)	1 (1.5%)	3 (5.7%)	0.001
**TEK**	4 (3.3%)	2 (3.0%)	2 (3.8%)	0.035
**XPO1**	4 (3.3%)	2 (3.0%)	2 (3.8%)	0.803
**ABL1**	3 (2.5%)	0 (0.0%)	3 (5.7%)	0.002
**AKT1**	3 (2.5%)	2 (3.0%)	1 (1.9%)	0.645
**AR**	3 (2.5%)	1 (1.5%)	2 (3.8%)	0.510
**AURKB**	3 (2.5%)	2 (3.0%)	1 (1.9%)	0.891
**BARD1**	3 (2.5%)	1 (1.5%)	2 (3.8%)	0.790
**BCOR**	3 (2.5%)	3 (4.5%)	0 (0.0%)	0.244
**CCND2**	3 (2.5%)	1 (1.5%)	2 (3.8%)	0.372
**CD79B**	3 (2.5%)	2 (3.0%)	1 (1.9%)	0.942
**CDC73**	3 (2.5%)	2 (3.0%)	1 (1.9%)	0.787
**CHEK2**	3 (2.5%)	1 (1.5%)	2 (3.8%)	0.296
**CTNNA1**	3 (2.5%)	1 (1.5%)	2 (3.8%)	0.005
**DDR1**	3 (2.5%)	2 (3.0%)	1 (1.9%)	0.052
**FGF23**	3 (2.5%)	1 (1.5%)	2 (3.8%)	0.372
**FGFR2**	3 (2.5%)	2 (3.0%)	1 (1.9%)	0.726
**FOXL2**	3 (2.5%)	2 (3.0%)	1 (1.9%)	0.772
**GSK3B**	3 (2.5%)	1 (1.5%)	2 (3.8%)	0.635
**HGF**	3 (2.5%)	2 (3.0%)	1 (1.9%)	0.480
**HNF1A**	3 (2.5%)	3 (4.5%)	0 (0.0%)	0.421
**IKBKE**	3 (2.5%)	1 (1.5%)	2 (3.8%)	0.092
**IKZF1**	3 (2.5%)	2 (3.0%)	1 (1.9%)	0.847
**JUN**	3 (2.5%)	2 (3.0%)	1 (1.9%)	0.561
**KEAP1**	3 (2.5%)	2 (3.0%)	1 (1.9%)	0.954
**MAP2K4**	3 (2.5%)	1 (1.5%)	2 (3.8%)	0.045
**MAPK1**	3 (2.5%)	2 (3.0%)	1 (1.9%)	0.717
**MEN1**	3 (2.5%)	3 (4.5%)	0 (0.0%)	0.235
**MLH1**	3 (2.5%)	2 (3.0%)	1 (1.9%)	0.742
**PDGFRB**	3 (2.5%)	1 (1.5%)	2 (3.8%)	0.076
**PIK3C2B**	3 (2.5%)	2 (3.0%)	1 (1.9%)	0.387
**PPARG**	3 (2.5%)	0 (0.0%)	3 (5.7%)	0.103
**PRDM1**	3 (2.5%)	2 (3.0%)	1 (1.9%)	0.929
**PRKAR1A**	3 (2.5%)	2 (3.0%)	1 (1.9%)	0.965
**RAC1**	3 (2.5%)	1 (1.5%)	2 (3.8%)	0.311
**RAD51B**	3 (2.5%)	2 (3.0%)	1 (1.9%)	0.962
**RAD52**	3 (2.5%)	1 (1.5%)	2 (3.8%)	0.372
**SRC**	3 (2.5%)	3 (4.5%)	0 (0.0%)	0.180

**VEGFA**	3 (2.5%)	3 (4.5%)	0 (0.0%)	0.802
**WT1**	3 (2.5%)	0 (0.0%)	3 (5.7%)	0.038
**AKT3**	2 (1.7%)	2 (3.0%)	0 (0.0%)	0.320
**ALOX12B**	2 (1.7%)	0 (0.0%)	2 (3.8%)	0.851
**BCL2L2**	2 (1.7%)	1 (1.5%)	1 (1.9%)	0.699
**CDH1**	2 (1.7%)	0 (0.0%)	2 (3.8%)	0.020
**CDK4**	2 (1.7%)	2 (3.0%)	0 (0.0%)	0.816
**CEBPA**	2 (1.7%)	0 (0.0%)	2 (3.8%)	0.420
**CSF3R**	2 (1.7%)	1 (1.5%)	1 (1.9%)	0.976
**CTNNB1**	2 (1.7%)	2 (3.0%)	0 (0.0%)	0.497
**CUL3**	2 (1.7%)	1 (1.5%)	1 (1.9%)	0.478
**DDR2**	2 (1.7%)	2 (3.0%)	0 (0.0%)	0.162
**EED**	2 (1.7%)	1 (1.5%)	1 (1.9%)	0.961
**ERG**	2 (1.7%)	1 (1.5%)	1 (1.9%)	0.990
**ESR1**	2 (1.7%)	0 (0.0%)	2 (3.8%)	0.030
**EZH2**	2 (1.7%)	1 (1.5%)	1 (1.9%)	0.505
**FAM46C**	2 (1.7%)	1 (1.5%)	1 (1.9%)	0.021
**FANCL**	2 (1.7%)	1 (1.5%)	1 (1.9%)	0.828
**FGFR4**	2 (1.7%)	1 (1.5%)	1 (1.9%)	0.136
**GABRA6**	2 (1.7%)	2 (3.0%)	0 (0.0%)	0.242
**GATA6**	2 (1.7%)	2 (3.0%)	0 (0.0%)	0.324
**GNAQ**	2 (1.7%)	0 (0.0%)	2 (3.8%)	0.134
**HDAC1**	2 (1.7%)	2 (3.0%)	0 (0.0%)	0.319
**IDH2**	2 (1.7%)	2 (3.0%)	0 (0.0%)	0.896
**JAK1**	2 (1.7%)	2 (3.0%)	0 (0.0%)	0.466
**MAP2K1**	2 (1.7%)	0 (0.0%)	2 (3.8%)	0.074
**MITF**	2 (1.7%)	0 (0.0%)	2 (3.8%)	0.005
**MPL**	2 (1.7%)	1 (1.5%)	1 (1.9%)	0.600
**MYD88**	2 (1.7%)	1 (1.5%)	1 (1.9%)	0.753
**NF2**	2 (1.7%)	2 (3.0%)	0 (0.0%)	0.521
**PAX5**	2 (1.7%)	0 (0.0%)	2 (3.8%)	1.71E-07
**PBRM1**	2 (1.7%)	1 (1.5%)	1 (1.9%)	0.874
**PDCD1**	2 (1.7%)	0 (0.0%)	2 (3.8%)	0.005
**PIK3R1**	2 (1.7%)	2 (3.0%)	0 (0.0%)	0.762
**PPP2R2A**	2 (1.7%)	1 (1.5%)	1 (1.9%)	0.636
**PTPN11**	2 (1.7%)	1 (1.5%)	1 (1.9%)	0.095
**RAF1**	2 (1.7%)	1 (1.5%)	1 (1.9%)	0.745
**RBM10**	2 (1.7%)	0 (0.0%)	2 (3.8%)	0.053
**SDHB**	2 (1.7%)	1 (1.5%)	1 (1.9%)	0.858
**SF3B1**	2 (1.7%)	2 (3.0%)	0 (0.0%)	0.275
**SMAD4**	2 (1.7%)	1 (1.5%)	1 (1.9%)	1.58E-14

**SMARCB1**	2 (1.7%)	1 (1.5%)	1 (1.9%)	0.298
**SMO**	2 (1.7%)	1 (1.5%)	1 (1.9%)	0.852
**SUFU**	2 (1.7%)	1 (1.5%)	1 (1.9%)	0.770
**SYK**	2 (1.7%)	2 (3.0%)	0 (0.0%)	0.405
**TYRO3**	2 (1.7%)	1 (1.5%)	1 (1.9%)	0.540
**WHSC1**	2 (1.7%)	2 (3.0%)	0 (0.0%)	0.313
**A1147P**	1 (0.8%)	0 (0.0%)	1 (1.9%)	0.685
**A512T**	1 (0.8%)	0 (0.0%)	1 (1.9%)	0.926
**AKT2**	1 (0.8%)	0 (0.0%)	1 (1.9%)	0.362
**ARID1**	1 (0.8%)	1 (1.5%)	0 (0.0%)	0.831
**BCL2**	1 (0.8%)	0 (0.0%)	1 (1.9%)	0.151
**BTG1**	1 (0.8%)	1 (1.5%)	0 (0.0%)	0.927
**CBFB**	1 (0.8%)	0 (0.0%)	1 (1.9%)	0.133
**CCND3**	1 (0.8%)	1 (1.5%)	0 (0.0%)	0.927
**CD79A**	1 (0.8%)	1 (1.5%)	0 (0.0%)	0.525
**CDK8**	1 (0.8%)	1 (1.5%)	0 (0.0%)	0.498
**D256Y**	1 (0.8%)	0 (0.0%)	1 (1.9%)	0.685
**DAXX**	1 (0.8%)	0 (0.0%)	1 (1.9%)	0.116
**EPBH1**	1 (0.8%)	1 (1.5%)	0 (0.0%)	0.394
**ERRFI1**	1 (0.8%)	1 (1.5%)	0 (0.0%)	0.449
**EZR**	1 (0.8%)	0 (0.0%)	1 (1.9%)	0.362
**FAM123B**	1 (0.8%)	1 (1.5%)	0 (0.0%)	0.489
**FG19**	1 (0.8%)	1 (1.5%)	0 (0.0%)	0.756
**FGF2**	1 (0.8%)	0 (0.0%)	1 (1.9%)	0.021
**FH**	1 (0.8%)	1 (1.5%)	0 (0.0%)	0.525
**FNACA**	1 (0.8%)	0 (0.0%)	1 (1.9%)	0.704
**G1160E**	1 (0.8%)	0 (0.0%)	1 (1.9%)	0.926
**G419R**	1 (0.8%)	0 (0.0%)	1 (1.9%)	0.685
**G45R**	1 (0.8%)	0 (0.0%)	1 (1.9%)	0.685
**G886S**	1 (0.8%)	0 (0.0%)	1 (1.9%)	0.926
**GATA4**	1 (0.8%)	1 (1.5%)	0 (0.0%)	0.470
**GTAT4**	1 (0.8%)	1 (1.5%)	0 (0.0%)	0.784
**ID3**	1 (0.8%)	1 (1.5%)	0 (0.0%)	0.359
**IDH1**	1 (0.8%)	0 (0.0%)	1 (1.9%)	0.467
**INK4B**	1 (0.8%)	1 (1.5%)	0 (0.0%)	0.560
**MDM4**	1 (0.8%)	1 (1.5%)	0 (0.0%)	0.460
**MEF2B**	1 (0.8%)	0 (0.0%)	1 (1.9%)	0.704
**MRE11A**	1 (0.8%)	0 (0.0%)	1 (1.9%)	0.362
**MYCL**	1 (0.8%)	0 (0.0%)	1 (1.9%)	0.560
**NOTH3**	1 (0.8%)	0 (0.0%)	1 (1.9%)	0.071
**NPM1**	1 (0.8%)	1 (1.5%)	0 (0.0%)	0.784

**NT5C2**	1 (0.8%)	0 (0.0%)	1 (1.9%)	0.903
**P10L**	1 (0.8%)	0 (0.0%)	1 (1.9%)	0.685
**P2RY8**	1 (0.8%)	1 (1.5%)	0 (0.0%)	0.285
**P484T**	1 (0.8%)	0 (0.0%)	1 (1.9%)	0.685
**PIM1**	1 (0.8%)	1 (1.5%)	0 (0.0%)	0.378
**Q53P**	1 (0.8%)	0 (0.0%)	1 (1.9%)	0.685
**QKI**	1 (0.8%)	1 (1.5%)	0 (0.0%)	0.831
**R363S**	1 (0.8%)	0 (0.0%)	1 (1.9%)	0.926
**R384I**	1 (0.8%)	0 (0.0%)	1 (1.9%)	0.926
**R401C**	1 (0.8%)	0 (0.0%)	1 (1.9%)	0.685
**R41W**	1 (0.8%)	0 (0.0%)	1 (1.9%)	0.685
**R98K**	1 (0.8%)	0 (0.0%)	1 (1.9%)	0.685
**RARA**	1 (0.8%)	1 (1.5%)	0 (0.0%)	0.831
**REL**	1 (0.8%)	1 (1.5%)	0 (0.0%)	0.870
**S670R**	1 (0.8%)	0 (0.0%)	1 (1.9%)	0.926
**SDHC**	1 (0.8%)	1 (1.5%)	0 (0.0%)	0.814
**SMAD2**	1 (0.8%)	1 (1.5%)	0 (0.0%)	0.654
**SPOP**	1 (0.8%)	1 (1.5%)	0 (0.0%)	0.927
**TMPRSS2**	1 (0.8%)	1 (1.5%)	0 (0.0%)	0.654
**TXB3**	1 (0.8%)	1 (1.5%)	0 (0.0%)	0.927
**U2AF1**	1 (0.8%)	1 (1.5%)	0 (0.0%)	0.489
**XRCC2**	1 (0.8%)	1 (1.5%)	0 (0.0%)	0.744

Supplementary Table 2. The somatic mutation rate of RMS and CRMS related genes observed in validation cohort.

Gene	Overall, n=160	Controls, n=94	Progressed, n=66	*P*	Asian, n=5
**RMS**					
SOX2	1 (0.6%)	1 (1.1%)	0 (0.0%)	0.398	1 (20.0%)
ATR	10 (6.2%)	5 (5.3%)	5 (7.6%)	0.771	-
JAK2	2 (1.2%)	0 (0.0%)	2 (3.0%)	**<0.001**	-
FANCG	-				
ZNF217	4 (2.5%)	3 (3.2%)	1 (1.5%)	0.542	-
EPHB4	3 (1.9%)	1 (1.1%)	2 (3.0%)	0.079	1 (20.0%)
MUTYH	2 (1.2%)	1 (1.1%)	1 (1.5%)	0.976	-
AXL	-				
CD22	3 (1.9%)	0 (0.0%)	3 (4.5%)	**0.003**	-
CDKN1A	-				
ERBB3	5 (3.1%)	1 (1.1%)	4 (6.1%)	**0.024**	-
RAD51D	1 (0.6%)	0 (0.0%)	1 (1.5%)	0.357	-
STAT3	4 (2.5%)	3 (3.2%)	1 (1.5%)	0.559	-
ABL1	3 (1.9%)	1 (1.1%)	2 (3.0%)	**0.040**	-
MITF	2 (1.2%)	2 (2.1%)	0 (0.0%)	0.445	-
PAX5	1 (0.6%)	1 (1.1%)	0 (0.0%)	0.473	-
**CRMS**					
MLL	-				
SETD2	5 (3.1%)	1 (1.1%)	4 (6.1%)	0.252	-
EP300	12 (7.5%)	7 (7.4%)	5 (7.6%)	0.928	1 (20.0%)
KMT2D	27 (16.9%)	17 (18.1%)	10 (15.2%)	0.560	1 (20.0%)
BRD4	3 (1.9%)	2 (2.1%)	1 (1.5%)	0.619	-
CREBBP	13 (8.1%)	6 (6.4%)	7 (10.6%)	0.270	-
DOT1L	8 (5.0%)	5 (5.3%)	3 (4.5%)	0.612	1 (20.0%)
TET2	3 (1.9%)	1 (1.1%)	2 (3.0%)	**0.045**	-
PRDM1	-				
WHSC1L1	1 (0.6%)	1 (1.1%)	0 (0.0%)	0.662	-
DNMT3A	5 (3.1%)	3 (3.2%)	2 (3.0%)	0.935	-
WHSC1	3 (1.9%)	1 (1.1%)	2 (3.0%)	0.428	-
HDAC1	-				
EZH2	2 (1.2%)	0 (0.0%)	2 (3.0%)	**0.009**	1 (20.0%)

P-value is estimated using log-rank test.

Supplementary Table 3. Cox regression analysis of relapse free survival (RFS) including clinicopathological factors, TMB, RMS and CRMS subgroup. HR, hazard ratio. CI, confidence interval.

Characteristics	Univariate		Multivariate (Model-A) [b]	
	HR (95% CI)	*P*	HR (95% CI)	*P*
**Clinicopathological factors**				
Age group (years) [a]				
<45	1.00		-	
>65	0.57 (0.21, 1.61)	0.291		
45-64	0.53 (0.21, 1.38)	0.194		
Sex				
Female	1.00		-	
Male	0.8 (0.29, 2.24)	0.678		
Location [a]				
HPC	1.00		1.00	
LC	2.13 (0.52, 8.75)	0.293	1.62 (0.30, 8.83)	0.579
OC	1.99 (0.80, 4.94)	0.138	2.63 (0.88, 7.82)	0.083
OPC	1.37 (0.48, 3.90)	0.551	2.49 (0.74, 8.41)	0.141
Grade				
Grade 1	1.00		-	
Grade 2	1.09 (0.56, 2.11)	0.796		
Grade 3	1.21 (0.55, 2.67)	0.638		
Margin [a]				
Free	1.00		1.00	
Not free	0.55 (0.23, 1.33)	0.187	0.67 (0.26, 1.73)	0.409
ENE [a]				
No	1.00		1.00	
Yes	1.92 (1.08, 3.40)	**0.026**	3.23 (1.55, 6.70)	**0.002**
LVI				
Negative	1.00		-	
Positive	1.45 (0.79, 2.65)	0.233		
PNI [a]				
Negative	1.00		1.00	
Positive	1.62 (0.91, 2.89)	0.100	1.30 (0.62, 2.73)	0.490
Stage				
Stage I	1.00		-	
Stage II	0.53 (0.10, 2.75)	0.454		
Stage III	0.52 (0.12, 2.18)	0.371		
Stage IV	0.78 (0.24, 2.52)	0.674		
CCRT response [a]				
Good (CR/PR/SD)	1.00		1.00	
Poor (PD)	7.40 (4.14, 13.2)	**<0.001**	6.70 (3.30, 13.6)	**<0.001**
**Somatic mutation profiles**				
TMB, mut/Mb				
Low (<3.78)	1.00		1.00	
High (≥3.78)	1.22 (0.67, 2.22)	0.508	2.29 (1.16, 4.53)	**0.017**
RMS				
Low (<1.135)	1.00		1.00	
High (≥1.135)	8.41 (4.44, 15.9)	**<0.001**	5.73 (2.61, 12.60)	**<0.001**
CRMS				
Low (<-0.62)	1.00		1.00	
High (≥-0.62)	5.82 (1.41, 24.0)	**0.015**	8.42 (1.77, 40.10)	**0.005**

[a] Clinicopathological factors with $p<0.2$ in univariate analysis results were included in multivariate

model.
[b] Variance inflation factors (VIF) of each factor included in multivariate model were computed, the included factors have no severe collinearity between each other’s.

Supplementary Table 4. Baseline characteristics of TCGA-HNSC validation cohort (n = 160) and matched validation cohort (n=60).

Characteristics	Overall, n = 160	Matched cohort, n = 60
**Clinicopathological factors**		
Age group (years)		
<45	17 (11%)	5 (8.3%)
>65	32 (20%)	11 (18%)
45-64	111 (69%)	44 (73%)
Sex		
Female	28 (18%)	4 (6.7%)
Male	132 (82%)	56 (93%)
Race		
Asian	5 (3.1%)	3 (5.0%)
Non-Asian	155 (97%)	57 (95%)
Stage		
Stage I	2 (1.3%)	2 (3.3%)
Stage II	5 (3.1%)	2 (3.3%)
Stage III	11 (6.9%)	8 (13%)
Stage IV	103 (64%)	32 (53%)
Unknown	39 (24%)	16 (27%)
CCRT response		
CR	69 (43%)	46 (77%)
PR	2 (1.3%)	2 (3.3%)
SD	2 (1.3%)	2 (3.3%)
PD	10 (6.2%)	10 (17%)
Unknown	77 (48%)	0 (0%)
**Survival outcome**		
Disease progressed	66 (41%)	27 (45%)
Deceased	58 (36%)	17 (28%)
**Somatic mutation profiles**		
TMB, mut/Mb		
Low (<3.78)	63 (39%)	19 (32%)
High (≥3.78)	97 (61%)	41 (68%)
RMS		
Low (<1.135)	146 (91%)	52 (87%)
High (≥1.135)	14 (8.8%)	8 (13%)
CRMS		
Low (<-0.62)	154 (96%)	58 (97%)
High (≥-0.62)	6 (3.8%)	2 (3.3%)

Supplementary Fig. 1. The prognostic risk predictive ability of (a) SOX2, (b) KLHL6, (c) ATR, (d) SOX2-ATR, (e) ATR-KLHL6, and (f) SOX2-KLHL6.

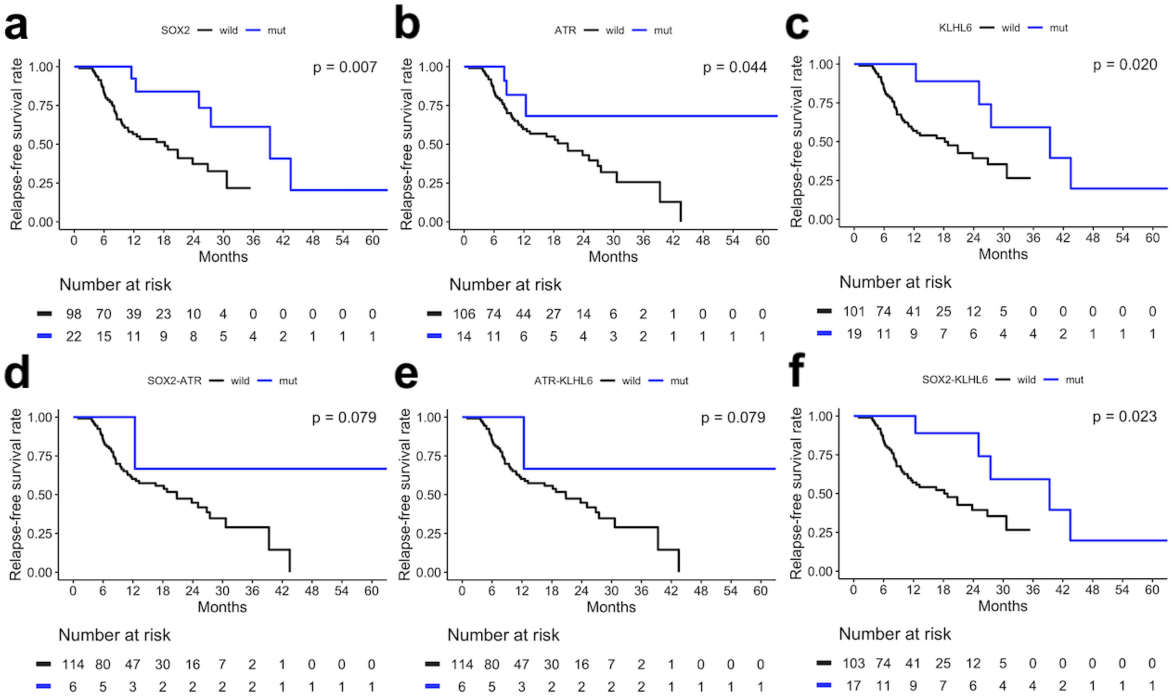

Supplementary Fig. 2. The prognostic risk predictive ability, satisfactory predictive performance, and boxplot of relapse mutation signature (RMS) and chromatin remodeling mutational signature (CRMS) in validation (a) overall TCGA-HNSC CCRT, (b) Asian, and (c) Non-Asian cohort. Kaplan-Meier plot for progression-free survival (PFS) according to (1) RMS, (2) CRMS, (3) RMS-CRMS subgroup, (4) satisfactory predictive performance between RMS, CRMS, RMS-CRMS determined using ROC, and boxplot of (5) RMS and (6) CRMS according to the progression status of each cohort were arranged from left to right.

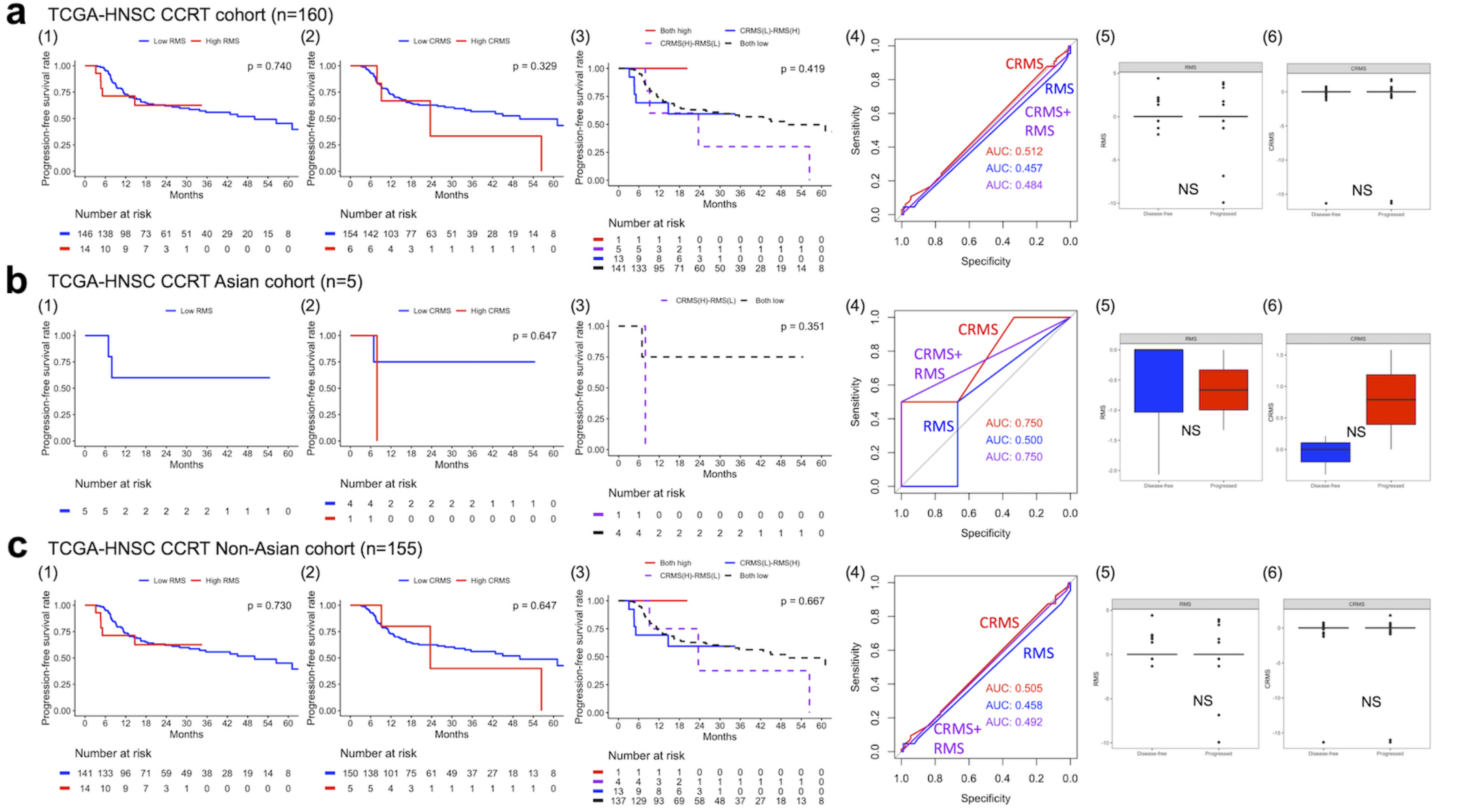

Supplement: Supplementary file 1 — Supplementary data [file 12276_2023_984_MOESM1_ESM.pdf]
